# Supplementary material for: CH vs. HC—Promiscuous Metal Sponges in Antimicrobial Peptides and Metallophores
Source: Molecules. 2023 May 9;28(10):3985. doi: 10.3390/molecules28103985 (PMC10221980; doi:10.3390/molecules28103985)

Supplementary Information

CH vs HC – promiscuous metal sponges in antimicrobial peptides and metallophores

Kinga Garstka[1], Valentyn Dzyhovskyi[1], Joanna Wątły[1], Kamila Stokowa-Sołtys[1], Jolanta Świątek-Kozłowska[2], Henryk Kozłowski[1,2], Miquel Barceló-Oliver[3], Denise Bellotti[1,4] and Magdalena Rowińska-Żyrek[1]*

1 Faculty of Chemistry, University of Wrocław, F. Joliot-Curie 14, 50-383 Wrocław, Poland

2 Faculty of Health Sciences, University of Opole, 68 Katowicka St., 45060 Opole, Poland

3 Department of Chemistry, University of Balearic Islands, Cra. de Valldemossa, km 7.5., 07122 Palma, Spain

4 Department of Chemical, Pharmaceutical and Agricultural Sciences, University of Ferrara, I-44121 Ferrara, Italy

* Correspondence: magdalena.rowinska-zyrek@uwr.edu.pl

Figure S1. Species distribution diagrams for the formation of A) Zn(II) complexes with the Ac-AHCA-NH_2 peptide; B) Ni(II) complexes with the Ac-AHCA-NH_2 peptide, C) Fe(II) complexes with the Ac-AHCA-NH_2 peptide; T=298 K; I=0.1 M; [L]=0.0005 M; M(II)/L molar ratio = 1 : 1

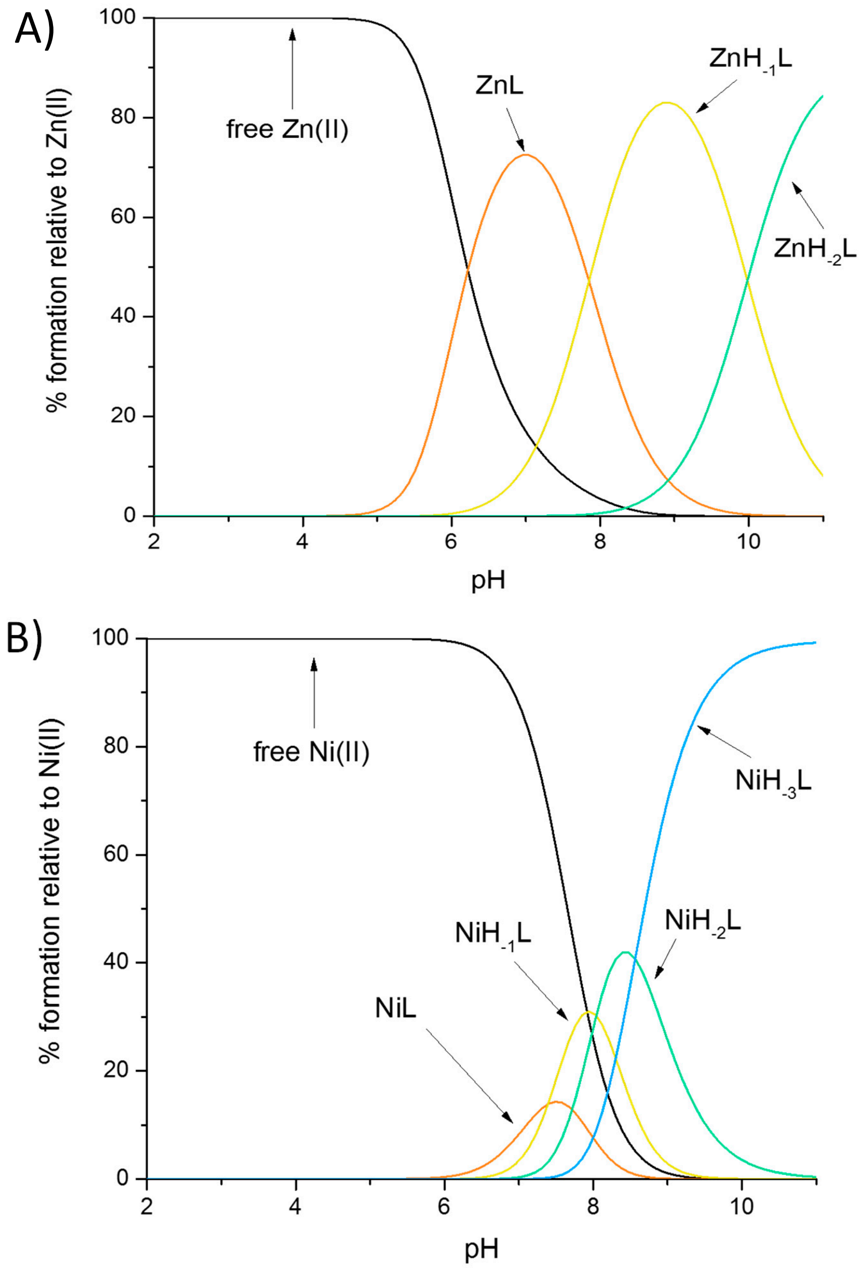

C)

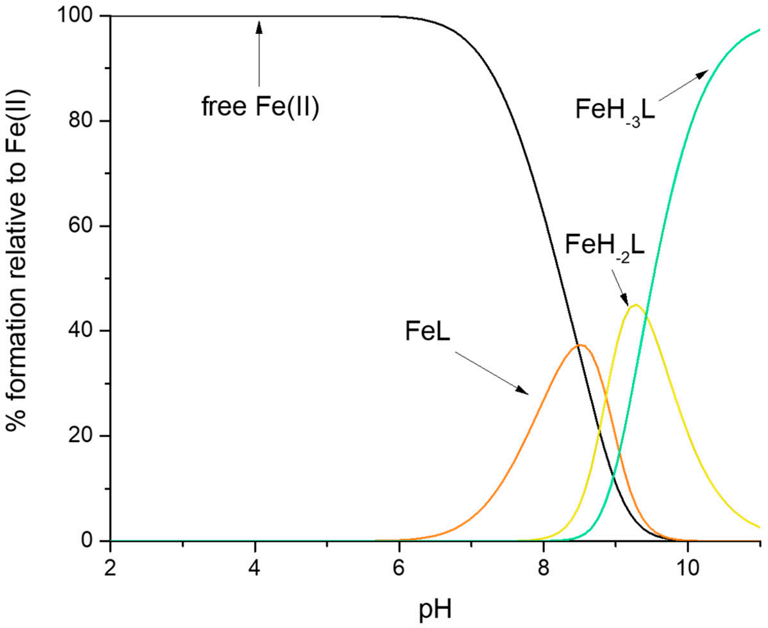

100
80
60
40
20
0
% formation relative to Fe(II)
2
4
6
8
10
pH
free Fe(II)
FeH-3L
FeH-2L
FeL

Figure S2. Distribution diagrams for the formation of A) Zn(II) complexes with the Ac-ACHA-NH_2 peptide; M(II)/L molar ratio = 1 : 1; B) Ni(II) complexes with the Ac-ACHA-NH_2 peptide; T=298 K; I=0.1 M; [L]=0.0005 M; M(II)/L molar ratio = 1 : 1; C) Fe(II) complexes with the Ac-AHCA-NH_2 peptide M(II)/L molar ratio = 1 : 1; T=298 K; I=0.1 M; [L]=0.0005 M.

A)

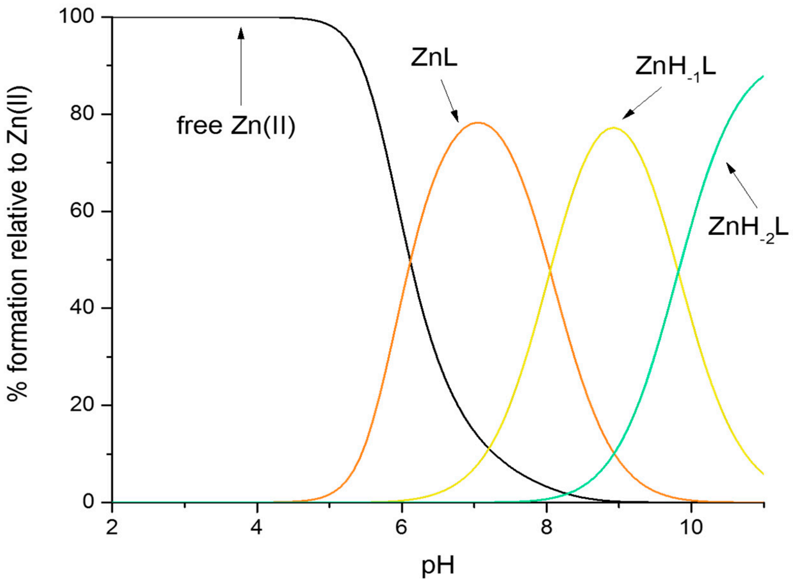

B)

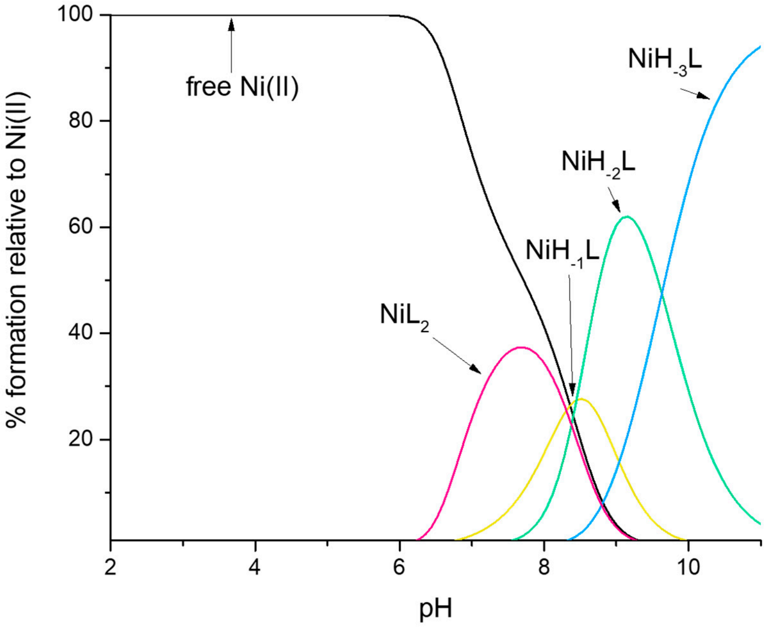

C)

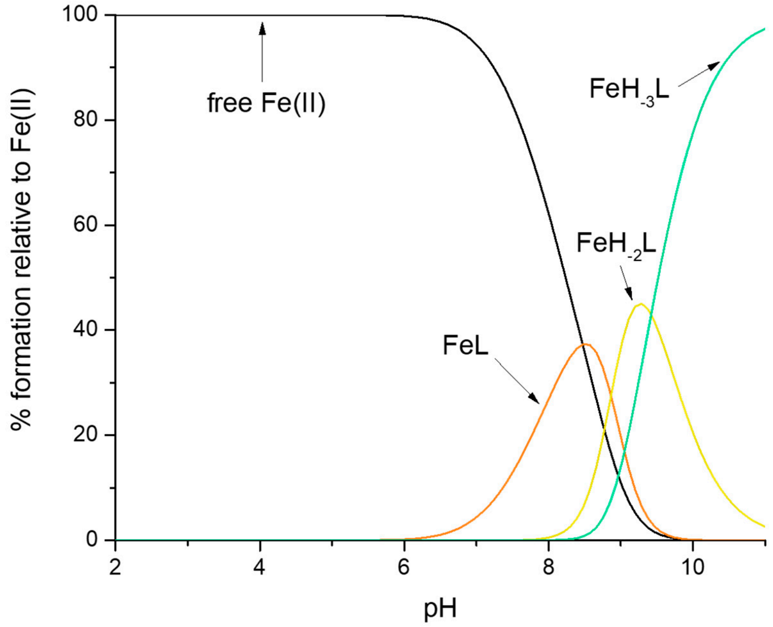

100
80
60
40
20
0
% formation relative to Fe(II)
2
4
6
8
10
pH
free Fe(II)
FeH-3L
FeH-2L
FeL

Figure S3. CD spectra of Ni(II) complexes with A) the Ac-AHCA-NH_2 peptide in the range 220-800 nm and pH range 2.5-10.5; T=298 K; optical path=1 cm; [L]=0.0005 M; M(II)/L = 1 : 1; B) the Ac-ACHA-NH_2 peptide in the range 220-800 nm and pH range 2.5-10.5; T=298 K; optical path=1 cm; [L]=0.0005 M; M(II)/L = 1 : 1.

A)

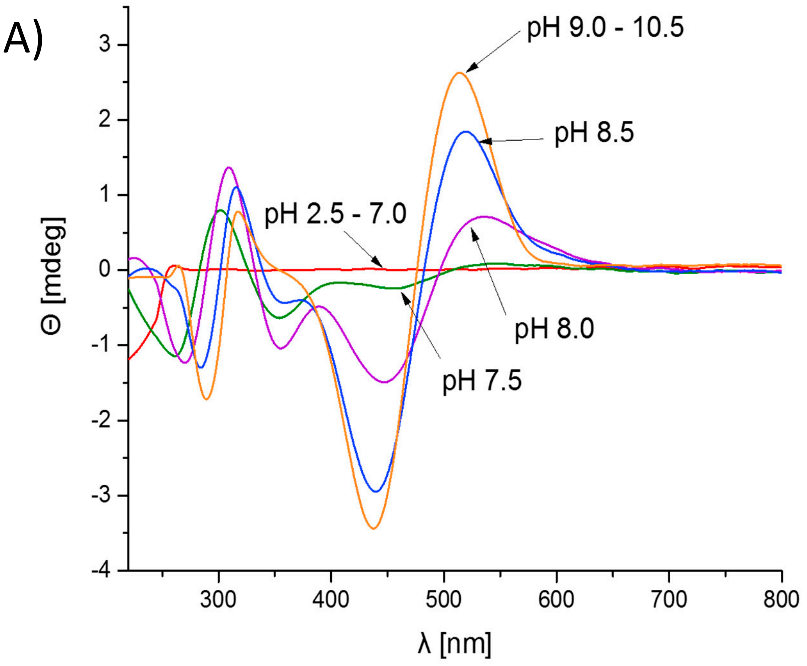

B)

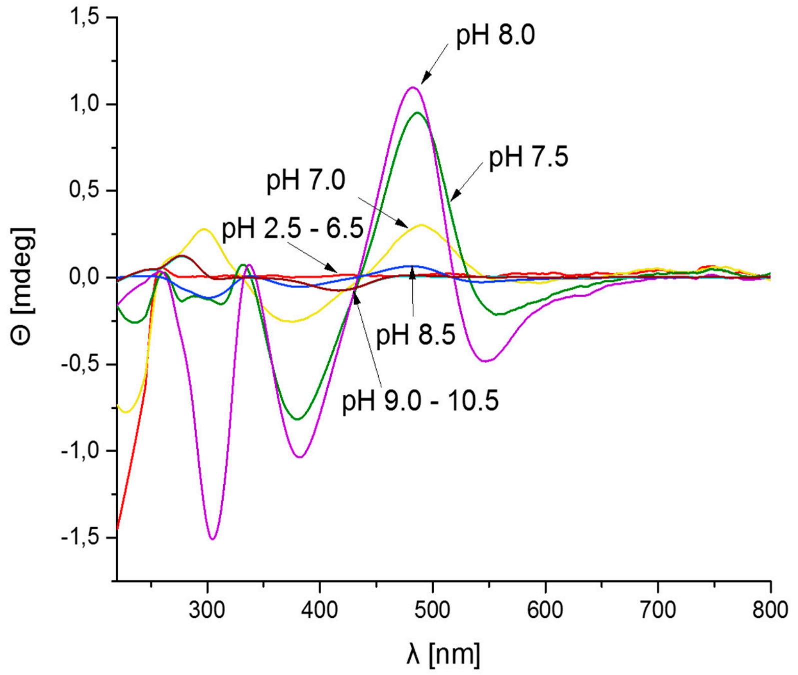

Figure S4. UV-Vis spectra of Ni(II) complexes with A) the Ac-AHCA-NH_2 peptide in the range 200-800 nm and pH range 2.5-10.5; T=298K; optical path=1 cm; [L]=0.0005 M; M(II)/L = 1 : 1; B) the Ac-ACHA-NH_2 peptide in the range 200-800 nm and pH range 2.5-10.5; T=298 K; optical path=1 cm; [L]=0.0005 M; M(II)/L = 1 : 1.

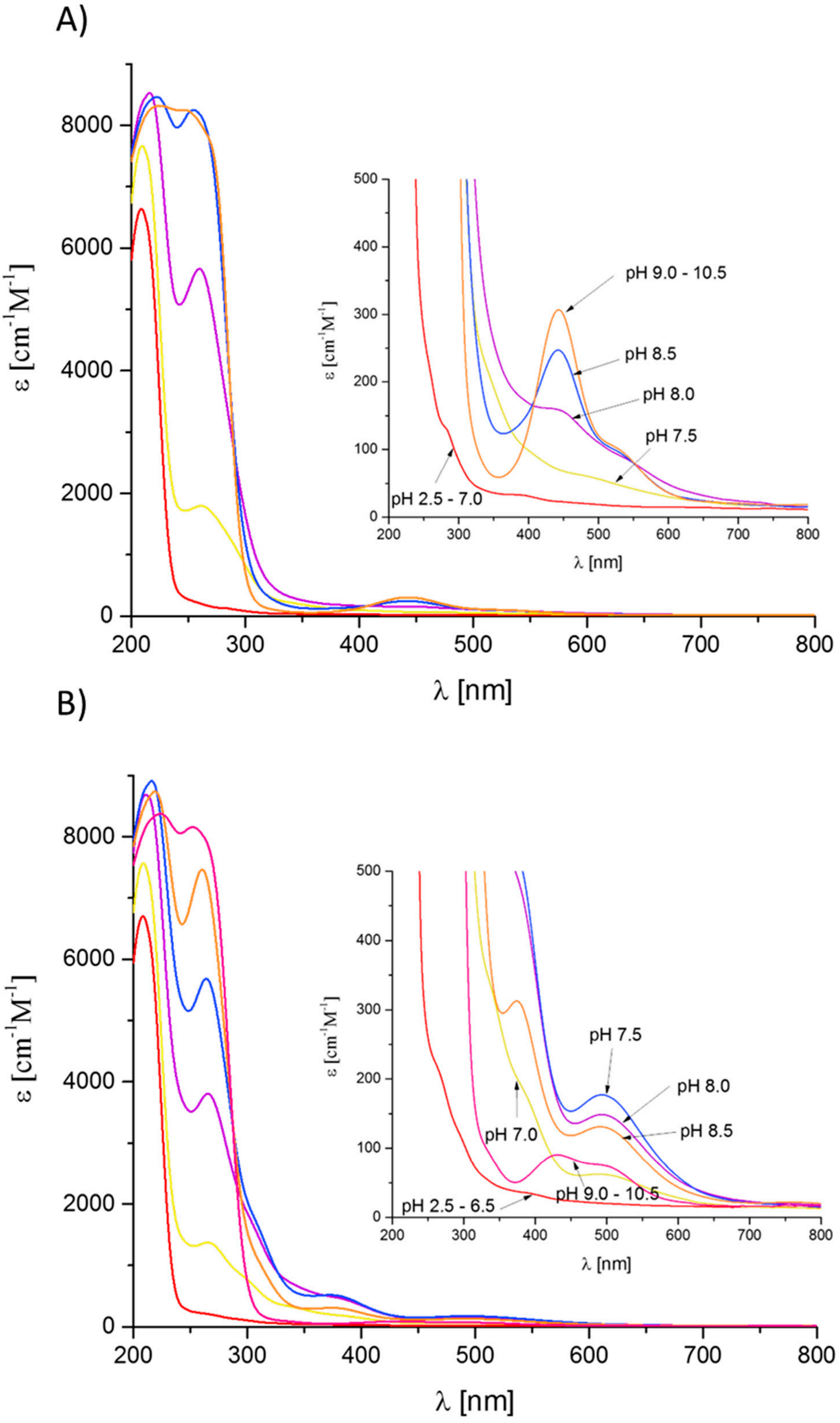

Supplement: Supplementary file 1 [file molecules-28-03985-s001.zip › molecules-2380111-supplementary.pdf]
